# Electronic properties of atomically thin MoS_2_ layers grown by physical vapour deposition: band structure and energy level alignment at layer/substrate interfaces†

**DOI:** 10.1039/c8ra00635k

**Published:** 2018-02-16

**Authors:** Fabio Bussolotti, Jainwei Chai, Ming Yang, Hiroyo Kawai, Zheng Zhang, Shijie Wang, Swee Liang Wong, Carlos Manzano, Yuli Huang, Dongzhi Chi, Kuan Eng Johnson Goh

**Affiliations:** Institute of Materials Research and Engineering, A*STAR (Agency for Science, Technology and Research) #08-03, 2 Fusionopolis Way, Innovis Singapore 138634 Singapore b.fabio@imre.a-star.edu.sg; Department of Physics, National University of Singapore 2 Science Drive 3 Singapore 117542 Singapore kejgoh@yahoo.com

## Abstract

We present an analysis of the electronic properties of an MoS_2_ monolayer (ML) and bilayer (BL) as-grown on a highly ordered pyrolytic graphite (HOPG) substrate by physical vapour deposition (PVD), using lab-based angle-resolved photoemission spectroscopy (ARPES) supported by scanning tunnelling microscopy (STM) and X-ray photoelectron spectroscopy (XPS) for morphology and elemental assessments, respectively. Despite the presence of multiple domains (causing in-plane rotational disorder) and structural defects, electronic band dispersions were clearly observed, reflecting the high density of electronic states along the high symmetry directions of MoS_2_ single crystal domains. In particular, the thickness dependent direct-to-indirect band gap transition previously reported only for MoS_2_ layers obtained by exfoliation or *via* epitaxial growth processes, was found to be also accessible in our PVD grown MoS_2_ samples. At the same time, electronic gap states were detected, and attributed mainly to structural defects in the 2D layers. Finally, we discuss and clarify the role of the electronic gap states and the interlayer coupling in controlling the energy level alignment at the MoS_2_/substrate interface.

## Abstract 2

The band structure of defective, rotationally disordered 2D TMDC layers is reported.

## Graphical abstract

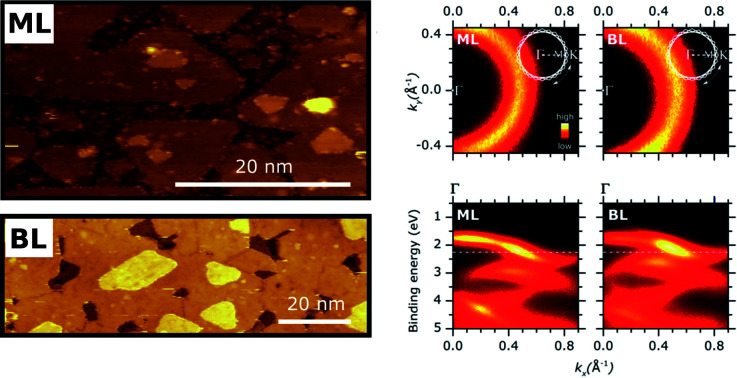

## 1. Introduction

Transition-metal dichalcogenides (TMDCs; MX_2_ where M = Mo or W and X = S, Se, or Te), are a wide class of layered semiconducting materials with promising functionalities for optoelectronic applications.^1^ The lattices of bulk TMDCs are formed by covalently bonded X–M–X hexagonal layers, which weakly bond with adjacent layers *via* van der Waals forces. At the monolayer limit (i) an indirect-to-direct band gap transition occurs due to the missing interlayer interaction^2–6^ which enhances the light absorption and the emission efficiency and (ii) a strong spin–orbit coupling combined with the broken inversion symmetry results in spin polarized bands,^7,8^ which makes TMDC monolayers suitable for spintronic applications.

High quality TMDCs layers with micrometer lateral size were first obtained from single crystals by a mechanical exfoliation technique, which is commonly used to isolate graphene layers.^9^ To meet the low cost wafer-scale fabrication requirements for industry adoption, large-scale deposition methods based on physical vapour deposition (PVD) and chemical vapour deposition (CVD) techniques were alternatively developed.^10,11^ A detailed characterization of the electronic properties of the as-grown TMDC layers at variance of the deposition conditions is critical for the device optimization, as they control the charge transport in the layer and at its interface with conductive electrodes. In this context, angle resolved photoemission spectroscopy (ARPES) studies have already proven to be useful for elucidating the electronic band structures of both exfoliated^12^ and large scale growth TMDC single crystal layers.^13–16^ Synchrotron-based ARPES facilities can achieve excellent energy and spatial resolutions due high intensity and collimated photon sources (≲100 μm of spot size^17,18^) but *in situ* 2D TMDCs growth cannot be generally provided which is detrimental for a proper optimization of the deposition process. In contrast, a lab-based ARPES may be installed in proximity to growth systems, but they are commonly affected by lower energy/spatial resolution due to their limited light source intensity and relatively large photon spot size (∼1 mm). In addition, structural defects (atomic vacancies within the lattice, grain boundaries, *etc.*) are generally introduced in the large scale growth process^19,20^ and can critically affect the electronic and optical properties of the layer. Despite their importance for applications, a detailed study of how the structural defects can impact on (i) the electronic band structures of as-grown TMDC layers and (ii) the charge injection at conductive interface is currently lacking.

In this paper, we report on the electronic properties of multi-domain MoS_2_ monolayer (ML) and bilayer (BL) grown by PVD on highly ordered pyrolytic graphite (HOPG) substrate using a lab-based ARPES system. The coexistence between in-plane rotational disorder and measurable band dispersion were demonstrated. The complex interplay between the defect related electronic gap states, and the interlayer coupling in determining the energy level alignment at MoS_2_/substrate interface were discussed.

## 2. Experimental

### 2.1 Sample preparation

HOPG substrates (HOPG, ZYA grade, SPI Supplies) were cleaved in air immediately before the introduction into the PVD growth chamber (pressure ∼ 10^−6^ mbar) and the surface was cleaned by *in situ* annealing at ∼1000 K for 10 min. The MoS_2_ layers (see schematic in Fig. S1 of ESI†) were deposited on the HOPG substrate by DC magnetron sputtering deposition technique from molybdenum (Mo) and sulphur (S) sources. The layer thickness was controlled by properly adjusting the total deposition time. Further details on the deposition technique were reported before.^10^ After deposition, the MoS_2_ layers were annealed *in situ* at ∼1000 K for 5 min to improve the layer crystallinity.

A high quality MoS_2_ natural crystal was also used as a reference for ARPES measurements. The crystal was cleaved in air before introduction into the preparation chamber of the ARPES system (pressure < 10^−9^ mbar).

### 2.2 Scanning tunnel microscopy (STM)

STM measurements were carried out in a custom-built multi-chamber ultra-high vacuum system housing an Omicron LT-STM, with a base pressure in the 10^−10^ mbar range. A chemically etched tungsten tip was used. The sample was degassed at 570 K for ∼12 h before STM analysis. The sample was kept at 77 K during all the measurements to obtain better resolution and all STM images were recorded in constant current mode.

### 2.3 X-ray photoelectron spectroscopy (XPS)

XPS measurements were performed at room temperature (298 K) in VG-ESCA lab 220i-XL using a monochromatic Al-K_α_ source (photon energy *hν* = 1486.7 eV). The samples were introduced from air in the UHV system (pressure < 10^−9^ mbar) and then measured as received. In the recorded kinetic energy range of Mo 3d and S 2p core levels (1100–1300 eV) a photoelectron mean free path of ∼5 nm (ref. 21) can be estimated, which is much larger than the typical thickness of surface adsorbate layers resulting from the air exposure (∼0.5 nm). In this context, the air exposure of the MoS_2_ samples had a limited impact on the measured XPS data, only resulting in a small attenuation of the XPS signal from the MoS_2_ layers. The binding energy scale was referred to the lowest binding energy component (which is attributed to sp^2^ C=C) in the C 1s spectra fixed at 284.5 eV, as measured in HOPG substrate, the energy resolution being set to 0.2 eV. XPS data were analysed by least square peak fitting procedure. Core level peaks were simulated by mixed Gaussian–Lorentzian functions. For the spin–orbit doublets, peak functions with same full width at half maximum (FWHM) were used, the spin orbit energy separation being set to 3.1 eV and 1.8 eV for Mo 3d and S 2p doublet, respectively.^22^ For each doublet, the relative intensity of the components (spin–orbit ratio) was fixed to 3 : 2 for Mo 3d and 2 : 1 for S 2p doublet.^22^

### 2.4 ARPES

The ARPES measurements were conducted at room temperature (298 K) in a custom-designed ARPES system, with a hemispherical electron analyser (SCIENTA DA30L) and monochromatized HeI_α_ (*hν* = 21.218 eV) radiation source (SCIENTA VUV5k). More details on the experimental setup can be found in ref. 23. A schematic description of the experimental geometry is shown in Fig. S2 of ESI.† Due to the special design of the analyser lens, data acquisition is possible in (i) “normal” ARPES mode, where the emission angle (*θ*_*x*_) is defined in the photoemission incidence plane and (ii) “deflection” ARPES mode, where full photoemission cone is accessible [*i.e.* both *θ*_*x*_ and *θ*_*y*_ are simultaneously measured (see Fig. S2 of ESI†)] within a range of ±15° with respect to the surface normal direction. Higher angular limits, to reach the boundaries of the SBZ, were obtained by proper adjustment of sample surface orientation, defined by *Θ*_*x*_ and *Θ*_*y*_ angles, with respect to the analyser lens entrance axis *z* (Fig. S2 of ESI†). More details on the experimental setup can be found in ref. 23. In both the ARPES acquisition modes the total energy resolution was set to 20 meV, the angular resolution being better than 0.2°. The binding energy scale was referred to the Fermi level (*E*_F_) as measured for a clean gold substrate.

All the ARPES data where acquired at 297 K. Before ARPES measurements the samples, as introduced from air in the UHV system, were annealed *in situ* (pressure < 10^−9^ mbar) at about 470 K for 12 h to remove surface adsorbates resulting from the air exposure.

### 2.5 Band structure calculations

First-principles band structure along the *ΓKM* and *ΓMΓ* direction of a MoS_2_ ML and BL were calculated by using the HSE06 hybrid functional with spin–orbit coupling as implemented in the Vienna *ab initio* simulation package (VASP).^24,25^ In calculating the band structure of ML and BL the experimental in plane lattice constant of 3.16 Å (ref. 26) was used. For BL the interlayer distance was set to 6.15 Å.^26^

## 3. Results and discussions

**Fig. 1:**
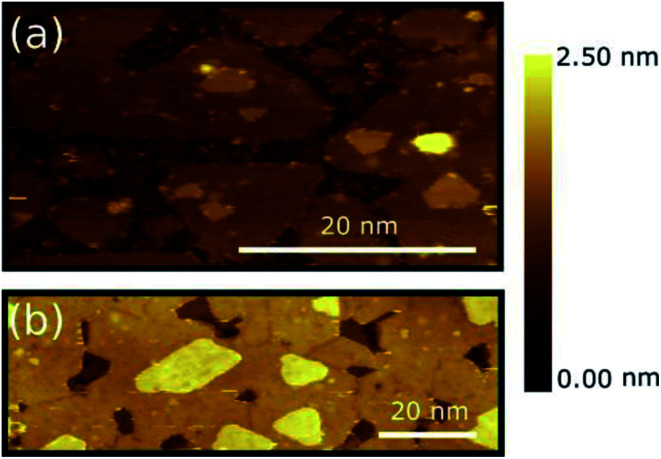
(a) STM topographic images of the MoS_2_ ML (*V*_tip_: 1.0 V, *I*_Tunnel_: 100 pA) (b) STM topographic images of the MoS_2_ BL (*V*_tip_: 1.0 V, *I*_Tunnel_: 50 pA). STM images were acquired at 77 K of sample temperature. The colour scale indicates the relative height variation from HOPG (darker area) to top MoS_2_ layers (brighter areas).

Fig. 1(a) and (b) show representative STM images of MoS_2_ layers deposited on HOPG, as obtained for ML [panel (a)] and BL [panel (b)] nominal layer thickness (see Methods). Large scale STM images are reported in Fig. S3 of the ESI.† The MoS_2_ ML deposition results in an almost complete coverage of the HOPG substrate [darker area in panel (a) and (b)], the layer mainly originating by the coalescence of multiple MoS_2_ grains and triangular islands with a typical lateral size < 50 nm. Smaller grains are also formed on top of some of the first layer islands clearly indicating that PVD deposited MoS_2_ grows in an island growth mode rather than in a layer-by-layer mode on HOPG substrate.^27^ A MoS_2_ interlayer separation of 6 ± 1 Å was estimated by extracting line profile across various step regions (see Fig. S3(c) of ESI†), with an MoS_2_–HOPG separation of 6 ± 1 Å, in good consistence with previous experimental reports.^20,26^ Small clusters of several nm in size in the flat areas between the grains are also clearly observed. Similar clusters were also found in other samples grown with comparable nominal thickness and can be tentatively identified with MoS_2_ particles by-product of the growth process that could not coalesce or bind to existing islands and that likely act as the MoS_2_ island precursors.

The sample with a MoS_2_ BL seems to undergo a similar growth process [see Fig. 1(b)]. Grain boundaries at the grain joints can also be clearly distinguished. Similar to the case of the MoS_2_ ML, the bilayer also shows triangular islands on top of the second layer indicating the early formation stages of a subsequent third layer. Additional Raman spectroscopy characterization of the MoS_2_ ML and BL is provided in Fig. S3(d) of ESI.†

A similar multi-domain 2D structures were reported for MoS_2_ layers deposited on various insulating amorphous substrates as SiO_2_ (ref. 10 and 28) where, however, a larger grain size was generally observed (∼20 μm of lateral size). The difference in domain sizes can be tentatively ascribed to (i) change in the growth conditions (substrate temperature, precursor, deposition techniques, *etc.*) and/or (ii) higher defect density on the HOPG substrate which can increase the number of possible of nucleation centres. Despite the difference in the grain size, the growth morphologies of PVD-grown MoS_2_ layers on HOPG substrate resembles those of TMDC layers deposited on insulating amorphous substrates as commonly required for applications.

**Fig. 2:**
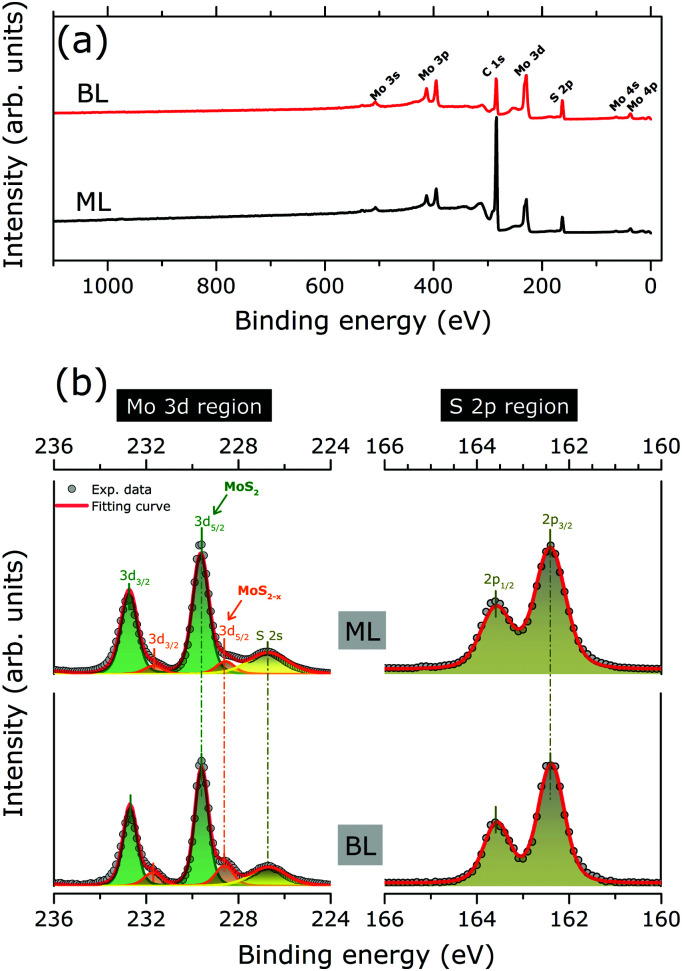
(a) XPS survey spectra of the as grown MoS_2_ ML and BL on HOPG (b) XPS data (grey full circles) of the as grown MoS_2_ ML and BL as acquired in the Mo3d (left panels) and S 2p (right panels) binding energy regions. Clear peak doublet structures resulting from the spin orbit-splitting of the Mo3d (3d_5/2_ and 3d_3/2_) and S 2p (2p_3/2_ and 2p_1/2_) core levels are observed. The S 2s core level is also observed in the Mo3d binding energy region. Two weak shoulders at the high binding energy sides of the main Mo3d peaks are visible, as related to the presence of MoS_2−*x*_ phase. The peak positions, as evaluated by least square fitting of the XPS data (see text for more details), are marked by vertical bars. The Mo3d doublet of MoS_2_ (MoS_2−*x*_) phase is indicated by green (orange) curves/areas. The S 2s (S 2p) components are indicated by bright (dark) yellows curves/area. For each region, the cumulative fitting curve is also indicated (red curves).

Fig. 2(a) shows the XPS survey spectra as acquired for MoS_2_ ML and BL, with Mo 3d and S 2p core level binding energy regions highlighted in Fig. 2(b). The results of the corresponding peak fitting analysis (see Section 2.3) are also included.

No significant changes in the core level peak positions and widths were observed with the increase of the MoS_2_ layer thickness. The peaks at 229.6 (163.6) and 237.7 (162.4) eV can be attributed to the Mo 3d_5/2_ (S 2p_3/2_) and Mo 3d_3/2_ (S 2p_1/2_) orbitals, respectively, in good agreement with previously reported binding energy values for the MoS_2_ layers.^13,29^ The S 2s component at 226.7 eV is also clearly visible.^10,29^ Finally, in both MoS_2_ ML and BL XPS data, two weak shoulders are visible at the low binding energy sides of the Mo3d main components, which can be reproduced by an additional Mo 3d spin–orbit doublet (Fig. 2). The binding energy positions of the doublet (228.4 for Mo 3d_5/2_, 225.3 eV for Mo 3d_3/2_) are consistent with the presence of under-coordinated Mo atoms of sub-stoichiometric MoS_2−*x*_, due to S-vacancies in MoS_2_ layers.^30^ The relative amount of under-coordinated Mo atoms with respect to the Mo atoms of the fully stoichiometric MoS_2_, were extracted from the ratio of the corresponding 3d doublets' area. A defect concentration of ∼10% and ∼20% was found for the MoS_2_ ML and MoS_2_ BL, respectively.

Structural defects resulting from atomic vacancies were reported to introduce electronic states in the energy gap between the valence and conduction bands of semiconducting materials.^31^ By acting as electron (hole) donor/acceptor centres, gap states may affect the *E*_F_ position in the energy gap and result in a p-type (*i.e. E*_F_ closer to the valence band edge) or n-type doping (*i.e. E*_F_ closer to the conduction band edge) of the semiconducting materials. In S-defective MoS_2_ layers an n-type doping was theoretically predicted^32^ with the S-vacancies introducing a high density of localized states close to conduction band edge.^33^ Moreover, the S-vacancies formation can be also accompanied by lattice distortion/reconstruction around each defect sites.^34^ Once laterally distributed in the 2D system, the S-related defects may alter, in principle, the lattice periodicity of the MoS_2_ layers with a consequent impact on the electronic band dispersion. With this in mind, the band structures on the PVD grown MoS_2_ ML and BL were carefully investigated by ARPES.

**Fig. 3:**
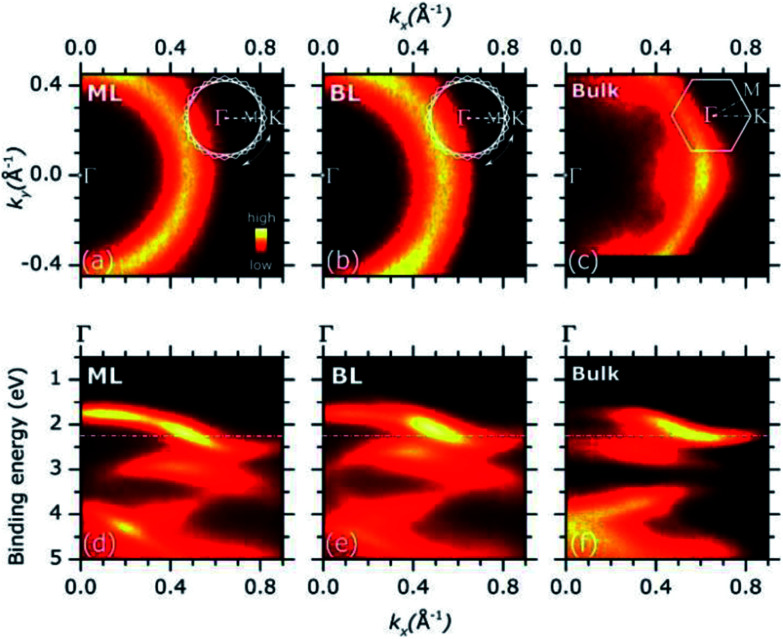
(a)–(c): Representative ARPES intensity constant energy map [binding energy = 2.30 eV, dash dotted line in panel (d)–(f)] of MoS_2_ ML (a), BL (b) and MoS_2_ bulk single crystal (c) as in the 2D (*k*_*x*_, *k*_*y*_) reciprocal space. The impact of the in-plane rotational disorder on the intensity pattern of the constant energy maps of the MoS_2_ ML and BL is schematically described in the insets with respect to the case of MoS_2_ bulk single crystal (see main text for details). (d)–(f): ARPES intensity of MoS_2_ ML (d), BL (e) as grown on HOPG substrates and MoS_2_ bulk single crystal (f) as a function of the binding energy and momentum component *k*_*x*_.

Representative ARPES constant energy maps (binding energy = 2.3 eV) in the 2D reciprocal space (*k*_*x*_, *k*_*y*_) are plotted in Fig. 3(a) (MoS_2_ ML) and (b) (MoS_2_ BL). The *k*_*x*_ and *k*_*y*_ represents the electron momentum component parallel to the substrate surface and measured in the *xz* (*k*_*x*_) and *yz* (*k*_*y*_) detector plane (see Fig. S2 of ESI†). The corresponding ARPES data of a MoS_2_ single crystal are also included in Fig. 3(c) for comparison, the *ΓK* high symmetry direction of the MoS_2_ SBZ lying in the *xz* detector plane [see inset in panel (c)].

The ARPES constant energy maps of MoS_2_ ML and BL [Fig. 3(a) and (b)] show circular intensity patterns around the *Γ* point of the SBZ (*k*_*x*_ = 0.00 Å^−1^, *k*_*y*_ = 0.00 Å^−1^). Similar circular-like patterns were obtained at different binding energy values and momentum ranges (data not shown). In MoS_2_ single crystal, a clear hexagonal-like ARPES map was observed [Fig. 3(c)]. In the ARPES measurements of single crystal materials the momentum distribution of the photoemission intensity was reported to reflect the symmetry of SBZ,^35,36^ as it was consistently found for the present MoS_2_ bulk sample [Fig. 3(c) and inset].

The circular-like ARPES maps observed for the PVD grown MoS_2_ ML and BL samples [Fig. 3(a) and (b)] originate from their multi-domain structure, evidenced by the STM analysis (see Fig. 1 and S3 of ESI†). In particular, the circular-like intensity patterns may be viewed as the result the incoherent superposition of many hexagonal patterns from each single crystal domain, reflecting the symmetry of the corresponding SBZs and their relative random orientation in the reciprocal space [see inset in Fig. 3(a) and (b)]. Our data suggest that the MoS_2_ ML and BL layer samples both consist of finite-size single crystalline domains which are much smaller than the analysis area (beam spot size ∼ 800 μm) with a complete in-plane rotational disorder.

The random orientation of the MoS_2_ ML and BL domains may partially originate from the rotational disorder of the HOPG substrate (lateral size of single crystal domain <100 μm (ref. 37)) which is transferred onto the 2D structure of the MoS_2_ layers due to the epitaxial growth conditions.^38^

Fig. 3(d) and (e) shows the ARPES intensity map of MoS_2_ ML (d) and BL (e) as a function of the binding energy and momentum component *k*_*x*_. For 2D layers with multi-domain structure, the band dispersion measured by ARPES along a given direction of the reciprocal space results from the superposition of the band dispersions of the various single crystal domains. In case of full in-plane rotational disorder, the band dispersion measured by ARPES is averaged over the entire SBZ of each single crystal domains which, in general, is expected to lead to no dispersion.

Despite the significant in-plane rotational disorder, giving the circular intensity map of Fig. 3(a) and (b), a clear band dispersion behaviour was observed in a wide energy/momentum range for both the ML and BL ARPES data [Fig. 3(d) and (e)]. The results qualitatively resemble the ARPES intensity map of MoS_2_ bulk single crystal as acquired along the *ΓK* high symmetry direction [Fig. 3(f)].

A similar coexistence of in-plane rotational disorder with measured band dispersion by ARPES were reported for HOPG.^37^ In particular, for any selected radial direction in the reciprocal space, ARPES mapping resulted from the superposition of the band dispersion of a single crystal graphite as measured along the high symmetry directions of the SBZ.^37^ The ARPES results were explained by the high density of electronic states in the various single crystal grains of HOPG, as mainly localized along the high symmetry directions of the corresponding SBZ *i.e.* van Hove singularities in the electronic density of states (DOS).^37^

**Fig. 4:**
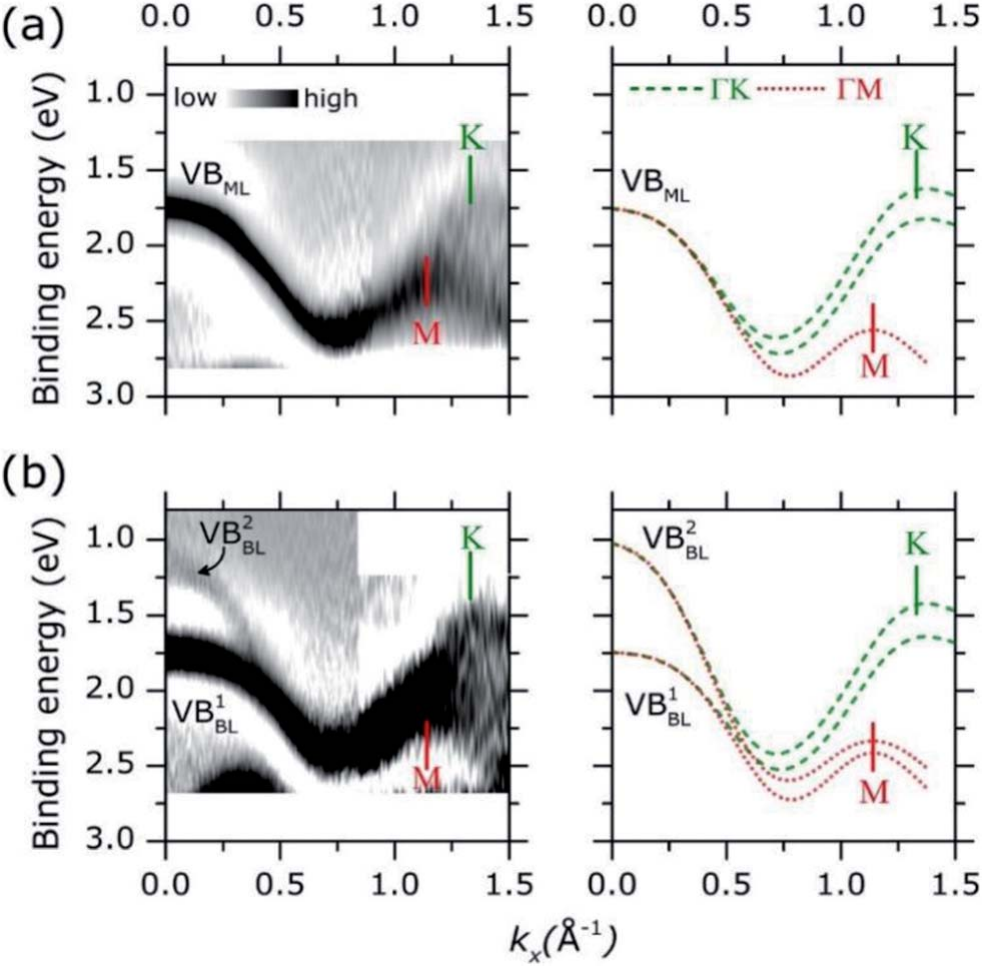
(a) Experimental (left panel) and theoretical (right panel) band dispersion along *ΓM* and *ΓK* direction of MoS_2_ ML on HOPG. Experimental band dispersion (darker area) were obtained by second derivative filter of corresponding ARPES data. Color intensity scale is indicated. Theoretical band dispersions along *ΓK* and *ΓM* direction are plotted as dashed green and dotted lines, respectively. Theoretical data were aligned at the binding energy position of the highest intense band at *Γ* point (*k*_*x*_ = 0.00 Å^−1^). The position of *K* and *M* high symmetry point in the reciprocal space is indicated by vertical bar. (b) Same as in panel (a) for the BL.

Adopting a similar treatment in our analysis, the experimental ARPES band dispersion of MoS_2_ ML and BL were compared with the calculated valence band structures along the *ΓK* and *ΓM* directions of the hexagonal SBZ. The results are shown in Fig. 4. The second derivative of the corresponding raw ARPES data intensity with respect to the energy (−d^2^*I*/d^2^*E*) is reported, in order to enhance the visibility of the experimental band dispersions.

In MoS_2_ ML [left panel in Fig. 4(a)], a single band (VB_ML_) is observed near the *Γ* point at ∼1.75 eV. As momentum increases the band dispersion gradually separates into two broad bands, their turning points being located at 1.14 Å^−1^ and 1.32 Å^−1^. By comparison with theoretical calculations [right panel of Fig. 4(a)], two (superimposed) band dispersions along the *ΓM* and *ΓK* directions can be clearly identified. The positions of the *M* (1.14 Å^−1^) and *K* (1.32 Å^−1^) points reflect the periodicity of the hexagonal SBZ in the reciprocal lattice [see Fig. S1 of the ESI†]. For the MoS_2_ BL [left panel in Fig. 4(b)] the experimental data can be similarly described, with a comparable level of accuracy, in terms of superimposed *ΓM* and *ΓK* band dispersion [right panel in Fig. 4(b)].

As for the HOPG case, the comparison between the experimental and theoretical band dispersions suggests a strong localization of the electronic density of states along the high symmetry directions of the SBZ of the MoS_2_ layers. This allows the ARPES measurements of the electronic band dispersions even in presence of in-plane rotational disorder.^37^ The results are further confirmed by the analysis ARPES spectra as acquired in a wider binding energy range, as shown in Fig. S4 of ESI.† More recently, the presence of van Hove singularities in the electronic density of states was theoretically suggested in order to explain the enhanced photo-absorption and hole–electron generation in 2D TMDCs layer.^39^

The energy broadening observed close to the high symmetry points of the experimental valence band dispersions in Fig. 4 can be ascribed to an “averaging” effect in introduced by the in-plane rotational disorder (see Fig. S5 of ESI for more discussion†) as similarly reported for HOPG samples.^37^ Because of the energy broadening, the small spin orbit splitting (∼150 meV) at *K* point cannot be resolved in our experimental data. Despite the broadening, however, it is rather apparent that the position of the valence band edge on MoS_2_ ML (*i.e.* at lowest binding energy) is located at *K* point (∼1.60 eV) instead of *Γ* (∼1.75 eV). This is highlighted in Fig. S6 of ESI† where a more detailed comparison of the MoS_2_ ML experimental and theoretical band dispersion near *Γ* and *K* point is presented.

As for the MoS_2_ BL, a remarkable result is that the valence band at *K* is located at higher binding energy with respect to the *Γ* point, where two band [VB^1^_BL_, VB^2^_BL_ in left panel of Fig. 4(b)] are observed. The different intensities of VB^1^_BL_ and VB^2^_BL_ band in the second derivative plot in Fig. 4(b) reflect their relative intensities in the MoS_2_ BL ARPES data [see Fig. S7 of ESI†]. Moreover, a decreasing of the overall ARPES signal at *Γ* point is observed in passing from ML to BL thickness [see comparison between Fig. 3(a) and (b) and Fig. S7 of ESI†], for almost completely vanishing in the bulk MoS_2_ single crystal [Fig. 3(c)]. This behaviour was previously observed in ARPES measurements on exfoliated MoS_2_ multilayer and related to matrix effect and multiple electron scattering during the photoemission process.^12^ Since the electronic states at *Γ* point are mainly derived from the Mo d_*z*^2^_ orbital in few-layer and bulk MoS_2_,^40^ the weak spectral intensity with respect to the ML case was explained as due to the slightly smaller in-plane lattice parameter in MoS_2_ multilayer and bulk,^7^ which allows for greater shielding by the S 2p orbitals.^41^

As shown in Fig. 4, the valence band dispersions of MoS_2_ ML and BL on HOPG along the *ΓK* direction are very well reproduced by the first principle calculation on MoS_2_ isolated layers. In view of our results and quite consistently with previous observations reported on similar systems,^15^ no significant impact of the HOPG dielectric screening on the valence band structure on deposited MoS_2_ layers appear to be present for our samples. More generally, any relevant substrate related effects are expected to affect differently the binding energy of the valence band states at *Γ* and *K* point, thus causing a valence band distortion with respect to the isolated layer case.^16^ The different impact of the substrate on the binding energy of *Γ* and *K* point reflects the difference in the spatial extension of the corresponding wave functions *e.g.* “∼out-of-plane” main orbital character (Mo3d_*z*^2^_) at Γ *vs.* main “∼in-plane” character at *K* point (Mo3d_*x*^2^−*y*^2^_, Mo3d_*xy*_).^40^ For the above reasons, the difference between theoretical and experimental bandwidth along the *ΓM* direction, where wave functions with out-of-plane orbital character also exist (Mo3d_*z*^2^_ at *M*) are unlikely due to substrate related effect, but may be attributed to other subtleties *e.g.* the details of DFT calculations. A significant distortion of the valence band along both the *ΓK* and *ΓM* direction would be observed otherwise. Further experimental and theoretical studies on MoS_2_ thin film with larger lateral size are currently in progress with the aim to clarify this issue.

The clear difference between the experimental band dispersion of MoS_2_ ML and BL (Fig. 4) supports the occurrence of a direct-to-indirect band transition with layer thickness, as seen in previous photoluminescence studies.^2,3^ This change in the electronic structure was ascribed to the interlayer interaction,^2,3^ which is responsible for the valence band splitting observed at the *Γ* point of the MoS_2_ BL [Fig. 4(b)] and the consequent transition of the valence band edge from the *K* point [ML, direct gap, Fig. 4(a)] to *Γ* point (BL, indirect gap). Similar evolution of the band structure as a function of number of layers was only reported, with comparable energy resolution and data quality, in synchrotron based ARPES studies of MoS_2_ single crystal layers as obtained by direct exfoliation^12^ or by direct epitaxial growth on conductive single crystal.^14^ Moreover, the relatively high concentration of S vacancies in ML (∼10%) and BL sample (∼20%) does not seem to have a significant impact on the measured band dispersion, which remain very similar to those of a free layers and BL. In the defect-related structural disorder is expected to simply cause an energy broadening of the ARPES spectra as mediated by electron scattering effect during the photoemission process.^35^ At the same time, a greater defect concentration near the domain boundaries can be suggested,^20^ thus leaving relatively unaffected the electronic band structure of the single crystal domain “inner” regions. In this context, our investigation demonstrate the possibility of fundamental studies on large scale growth TMDCs layers, as generally produced for applications, and whose growth morphology and structural properties (in plane rotational disorder, defects) were commonly assumed to hinder important details of the electronic band structures so limiting the ARPES studies to high quality single crystal TMDCs layers.

**Fig. 5:**
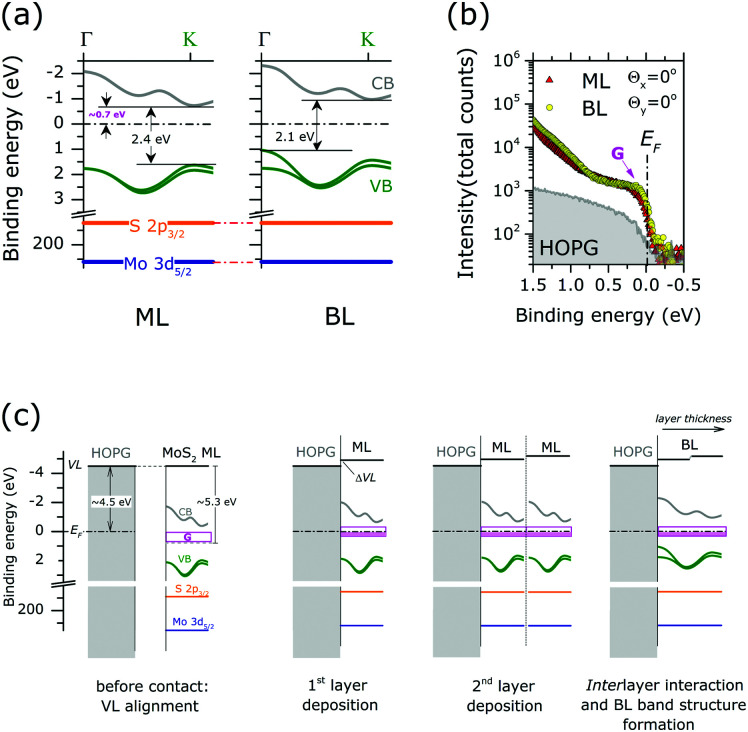
(a) Energy level diagram for the MoS_2_ ML (left) and BL (right). The calculated valence band (VB) and conduction band (CB) dispersion along *ΓK* direction are reported (see the figure). Mo 3d_5/2_ and S 2p_3/2_ binding energy positions of stoichiometric MoS_2_ are also indicated. The energy positions of the calculated CBs of the MoS_2_ ML and BL were rigidly shifted to reproduce the experimental gap value, as extracted from STS data of ref. 20 (b) angle integrated photoemission spectra (−15° ≤ *θ*_*x*_ ≤ 15°, −1° ≤ *θ*_*y*_ ≤ 1°) of MoS_2_ ML, MoS_2_ BL and HOPG bare substrate in the Fermi level (*E*_F_) binding energy range as acquired at normal emission condition (*Θ*_*x*_ = 0°, *Θ*_*y*_ = 0°). The intensity is reported in log scale to highlight minor variation of the photoemission signal. A clear increase in the density of gap state at *E*_F_(*G*) is observed upon MoS_2_ layers growth. Note the tailing of the ML and BL peak contributing to the intensity in the energy gap (>0.75 eV from *E*_F_) (c) schematic illustration of the energy level alignment mechanism at ML and BL interface with HOPG substrate. Shaded pink areas represent occupied gap states.

Finally, the energy level alignment of ML and BL at the interface with HOPG is discussed, with the support of the results of the previous ARPES and XPS results. Fig. 5(a) shows the energy diagram in the MoS_2_ ML and BL. The theoretical band dispersions along the *ΓK* directions were included, as they well reproduce (difference < 0.1 eV) the valence band edge positions at *Γ* and *K* point. The Mo 3d_5/2_ and S 2p_3/2_ core level position (as in Fig. 2) are also indicated. The energy position of the calculated conduction bands along the *ΓK* direction of the MoS_2_ ML and BL were rigidly shifted to reproduce the experimental gap values, as extracted from scanning tunnel spectroscopy measurements on MoS_2_ layers deposited on HOPG substrate.^20^

In MoS_2_ ML the *E*_F_ position in the (direct) energy gap lies at ∼0.7 eV from the conduction band indicating that the MoS_2_ layer is strongly n-doped. With respect to ML case the *E*_F_ position in the BL is located closer to the center of the (indirect) energy gap of the MoS_2_ BL, while no change in the core level binding energy position and FWHM is observed.

N-type doping of MoS_2_ ML was previously reported in ARPES studies of exfoliated^12^ and CVD-grown ML.^42^ The ML doping is likely to be ascribed to the high density of S vacancies, as evidenced in our XPS analysis (see Fig. 2). In particular, S-vacancies in MoS_2_ ML were theoretically predicted to introduce localized gap states at 0.6–0.7 eV from the conduction band edge.^33^ This suggests that the *E*_F_ position in the energy gap of MoS_2_ ML is pinned by the high density of gap states introduced by S vacancies in the layers. The existence of pinning gap states in the MoS_2_ ML is confirmed by a detailed ARPES study in the *E*_F_ binding energy region [Fig. 5(b)]. After MoS_2_ ML deposition on HOPG, a clear increase in the photoemission signal at the *E*_F_ is observed, which reflects the distribution of localized gap states (indicated with *G* in Fig. 5(b)) introduced by S vacancies in the 2D lattice structure. Interestingly, a comparable density of gap states at *E*_F_ is also detected in the MoS_2_ BL. This is in apparent contradiction with the results on the XPS analysis assigning a higher S-vacancies concentration (20% *vs.* 10%) to the MoS_2_ BL. This observation can be rationalized by considering the different surface sensitivity of the two photoemission based techniques. In the *E*_F_ region, the measured kinetic energy (∼17 eV) by ARPES corresponds to an electron mean free path of ∼3 Å.^21^ This value is much smaller with respect to the interlayer distance in MoS_2_ BL (6.15 Å). Because of the high surface sensitivity of the ARPES measurements, the photoemission signal at the *E*_F_ of MoS_2_ BL [Fig. 5(b)] mainly reflects the defect densities and related n-doping level of the outer layer at the interface with the vacuum, which is comparable to that of the MoS_2_ ML case. The excess of defect detected by XPS measurement (electron mean free path ∼5 nm (ref. )) in the BL sample may be related to S-vacancies introduced in the inner MoS_2_ layer during the top layer growth.

The above information contributes to clarify the mechanism of the energy level alignment at the interface with the HOPG in both MoS_2_ ML and BL system, as schematically illustrated in Fig. 5(c). In the energy level diagrams of Fig. 5(c) a work function value of 4.5 eV and 5.3 eV was respectively assumed for the HOPG^43^ and freestanding undoped MoS_2_ ML.^44^

Once the ML is in contact with HOPG substrate (*i.e.* 1^st^ layer deposition) interfacial charge transfers occurs to establish thermodynamic equilibrium, *i.e. E*_F_ alignment at the interface. At thermodynamic equilibrium, the position of the *E*_F_ in the energy gap on of MoS_2_ layer (0.7 eV from the conduction band edge) is entirely determined by the pinning gap states density (*G*) introduced by S-vacancies. This may result in vacuum level (VL) misalignment (ΔVL) and may be related to a dipole formation at the MoS_2_ ML/HOPG interface [Fig. 5(c)].

When an additional freestanding ML with comparable doping level is put in contact with ML/HOPG system (2^nd^ layer deposition) further charge transfer can occur at the interface between the MLs to reach thermodynamic equilibrium. An additional dipole can also result, reflecting the amount of charge transfer required to reach the Fermi level alignment across the layer. However, due to the pinning condition induced by the density of gap state, no change in the core level binding energies is expected upon film thickness increase, in agreement with our XPS measurements. Once thermodynamic equilibrium is established the interlayer interaction results in the final BL valence band structure, determining the observed position of the *E*_F_ in the indirect energy gap.

It is worth to note in passing that the substrate work function and MoS_2_ gap state density in each layer only affects the amount of charge transfer required for establishing the thermodynamic equilibrium across the various MoS_2_ layers. A lower density of gap state (*i.e.* no pinning condition) in each MoS_2_ layer may result, for example, in a gradual shift of the core level position as a function of thickness, as reported in previous XPS study on MoS_2_ multilayers.^45^ Moreover, in case of different doping levels among adjacent MoS_2_ layers, an energy offset is expected at the layer/layer interface. As the valence band edge position in each layer is different, this can also affect the interlayer interaction and the valence band structure of the multilayer. The above model suggests a possible strategy for controlling the electronic properties of TMDC multilayers. Further experimental and theoretical studies on this issue are currently in being pursued.

## 4. Conclusions

We have presented a detailed investigation of the electronic properties of atomically thin MoS_2_ layers, deposited on HOPG substrate by physical vapor deposition technique. A multi-domain structure was first evidenced in MoS_2_ ML and BL by detailed STM analysis. XPS investigation suggests, for both samples, a relatively high concentration of S-vacancies. Despite the randomly oriented multi-domain structure and defect concentrations, a clear band structure was extracted by ARPES. Also the coexistence of in-plane rotational disorder and a measurable band dispersion was demonstrated suggesting a high density of electronic states along high symmetry directions of each single crystal domain. In particular, our ARPES data provide sufficient energy resolution to demonstrate the expected direct-to-indirect band gap transition from ML to BL even in these somewhat imperfect grown layers. These results show that a lab-based ARPES system, such as ours, could readily provide meaningful fundamental investigations of the electronic band structure of large-area grown multi-domain 2D layers of TMDCs. This is a significant development given that the current challenge to reproducibly grow large-area 2D TMDCs is contingent upon the ability to readily characterize their properties so as to provide the needed frequent feedback for tuning the growth processes. The availability of a lab-based ARPES system (as demonstrated here) would greatly mitigate the longer lead-times to obtain measurements from significantly more costly synchrotron ARPES facilities. This would provide the much needed analysis for determining the layer electronic properties at site in a timely manner. As an example, the complex interplay between the defect related density of electronic states and interlayer interaction in determining the final position of the *E*_F_ in the energy gap of TMDCs films was discussed and clarified for our PVD grown large-area samples. These results suggest a strategy for tuning the interlayer interaction and consequently the band structure of TMDCs multilayer structure, which in turn can affect related optoelectronic applications.

## Conflicts of interest

There are no conflicts of interest to declare.

## Supplementary Material

RA-008-C8RA00635K-s001
