# Supplementary material for: Electronic properties of atomically thin MoS2 layers grown by physical vapour deposition: band structure and energy level alignment at layer/substrate interfaces
Source: RSC Adv. 2018 Feb 16;8(14):7744–52. doi: 10.1039/c8ra00635k (PMC9078486; doi:10.1039/c8ra00635k)
Supplement: RA-008-C8RA00635K-s001 [file RA-008-C8RA00635K-s001.pdf]

Electronic Supplementary Material (ESI) for RSC Advances.
This journal is © The Royal Society of Chemistry 2018

Supplemental Information

To

Electronic properties of atomically thin MoS_2 layers grown by physical vapour deposition: band structure and energy level alignment at layer/substrate interface

F. Bussolotti[*,1], J. W. Chai[1], M. Yang[1], H. Kawai[1], Z. Zhang[1], S. J. Wang[1], S. L. Wong[1], C. Manzano[1], Y. L. Huang[1], D. Z. Chi[1] and K. E. J. Goh[#1,2]

[1]*Institute of Materials Research and Engineering, A*STAR (Agency for Science, Technology and Research), #08-03, 2 Fusionopolis Way, Innovis, Singapore 138634,*

[2]*Department of Physics, National University of Singapore, 2 Science Drive 3, Singapore 117542, Singapore*

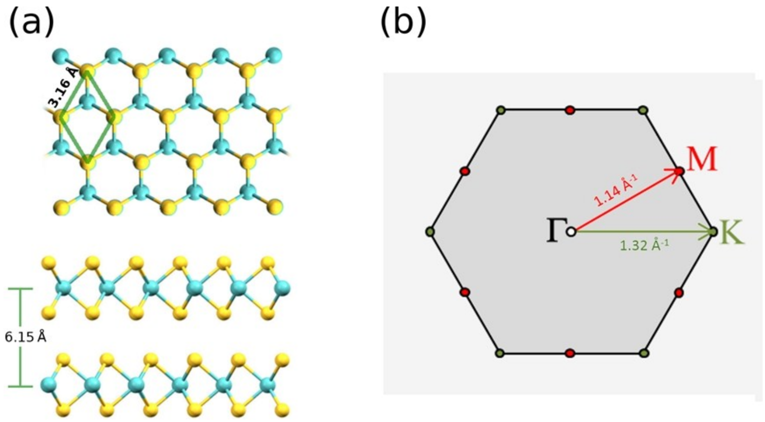

Figure S1. (a) Schematic 2D structure of MoS_2 ML (upper panel). The unit cell and corresponding lattice parameter are indicated. The side view of MoS_2 BL (2H structure) with an interlayer separation of 6.15 Å is also illustrated (lower panel) (b) 2D Surface Brillouin Zone of MoS_2 layer. The high symmetry ΓK and ΓM directions are indicated.

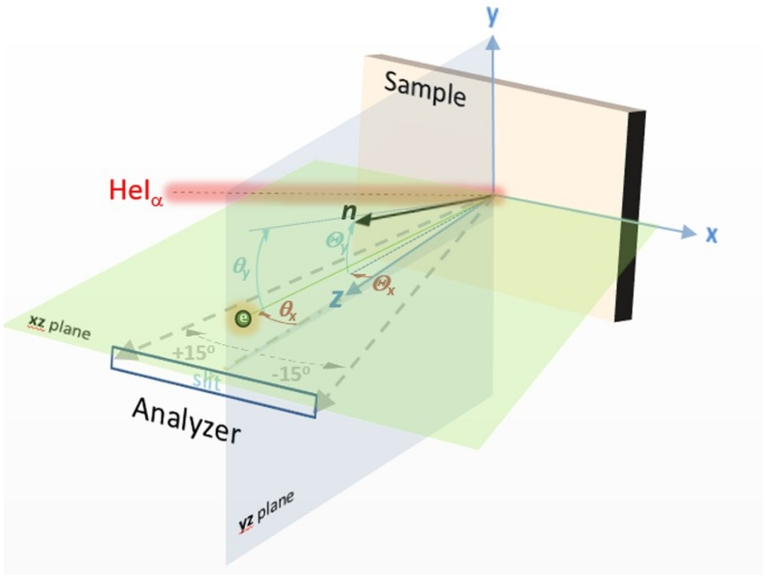

Figure S2. Schematic description of the ARPES experimental geometry (see main text for more details)

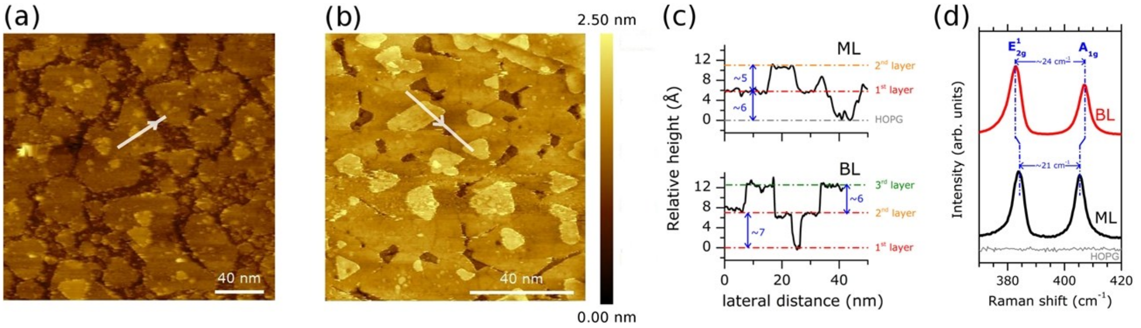

Figure S3. (a)-(b). STM topographic images of the MoS_2 ML (V_{tip}: 1.0 V, I_{Tunnel}: 100 pA) (a) and BL (V_{tip}: 1.0 V, I_{Tunnel}: 50 pA) (b) as acquired at 77 K. The colour scale indicate relative height variation from HOPG (darker area) to top MoS_2 layers (brighter areas) (c) Height line profile for ML and BL Data were acquired along the line (scanning direction indicated by arrow) in panel (a) and (b), respectively (c) Raman spectra of MoS_2 ML and BL in the range of the E^1_{2g} (in-plane) and A_{2g} (out of plane) vibration mode. Raman data of the bare HOPG substrate were also included for comparison purpose. The Raman spectra was obtained at room temperature (298 K) with 488 nm laser excitation using a Horiba Jobin Yvon LabRAM HR800 micro-Raman spectrometer. The Raman peak frequency difference in the ML (~21 cm^{-1}) is consistent with previous observation on CVD grown MoS_2 ML [S1-S2]. As discussed in Refs. S2, S3 the presence of defects [S3] or adsorbate material [S2] on the layer, as commonly introduced by large scale deposition methods, can affect the frequency of Raman vibration modes. This generally results in a larger peak frequency separation with respect to the value measured for high quality MoS_2 ML obtained by exfoliation from single crystal (~18~19 cm^{-1} [S4]). The increasing in the Raman peak energy separation from 21 cm^{-1} (ML) to 24 cm^{-1} (BL) is consistent with the increase of the layer number [S4].

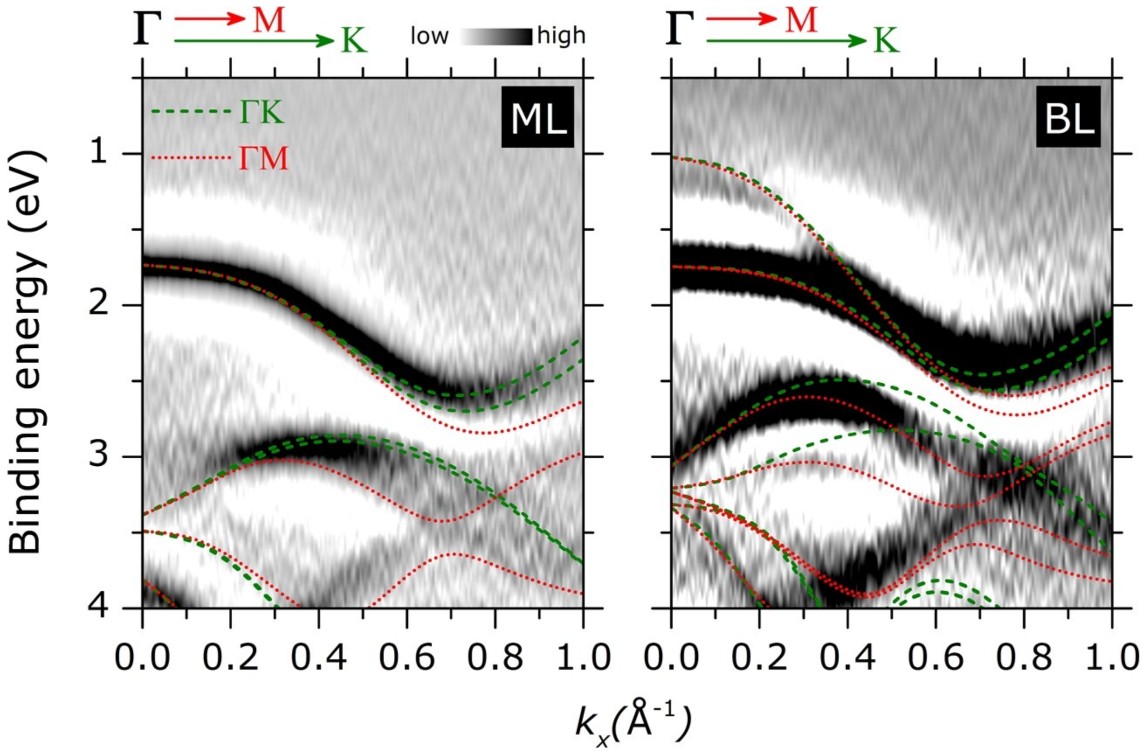

Figure S4. Second-derivative ARPES intensity map of MoS_2 ML (left panel) and BL (right panel) on HOPG, as obtained from the corresponding ARPES data in Figs. 3(a)-(b). Second derivative filter was applied to allow a clearer view of the ARPES dispersion. Intensity scale is indicated the left panel. Theoretical band dispersions along ΓK and ΓM direction are plotted as dashed green and dotted lines, respectively. Theoretical data were aligned at the binding energy position of the highest intense band at Γ point. It is worth to note in passing that the difference between the experimental and theoretical band dispersion as reported in the present work as well as in Figure 34 of the main text (i) reflects the well-known limit of DFT calculations in reproducing the energy band dispersion of material (Refs. 24,25 in the main text) and (ii) are fully in accord with the well-accepted protocol of analysis in previous reports that combined ARPES and DFT investigation on similar 2D systems (Ref. 12 of the main text).

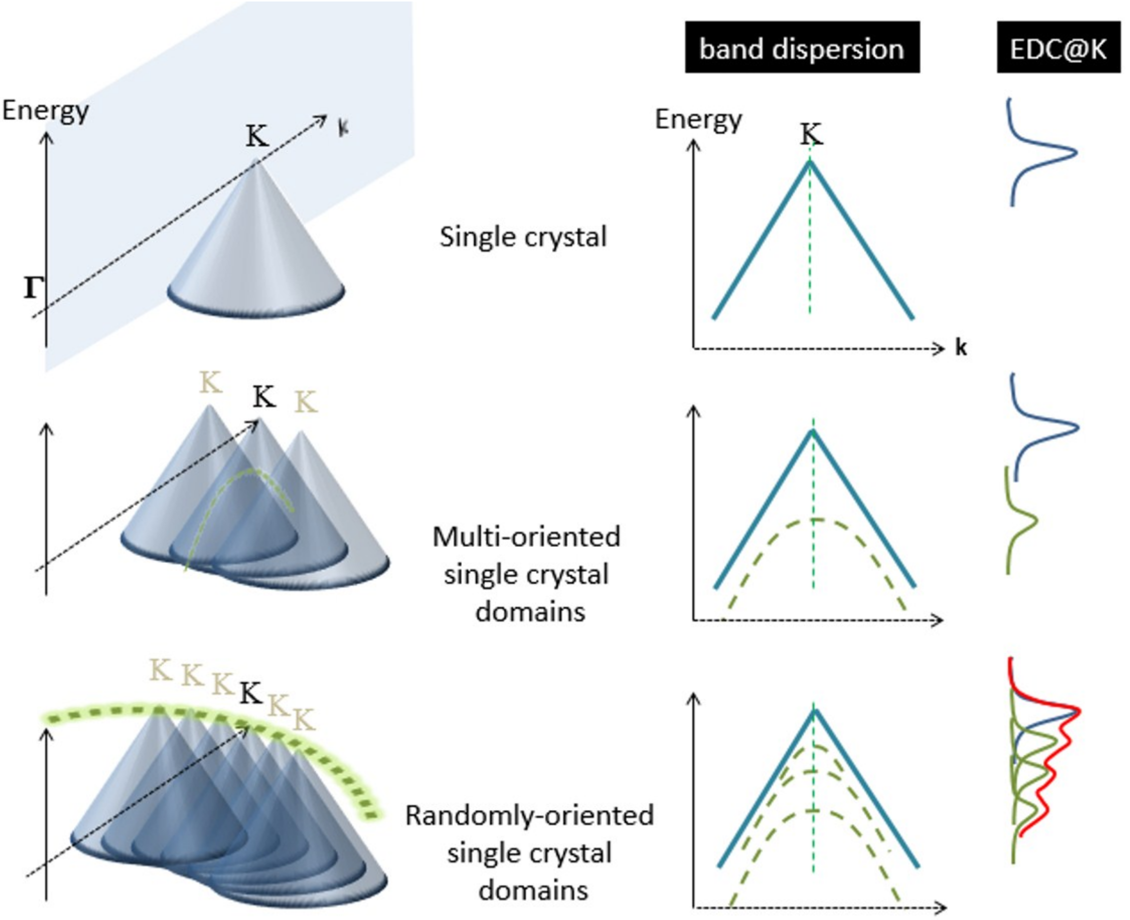

Figure S5. Qualitative description of the impact of the in-plane rotational disorder on the measured band dispersion at high symmetry point of the SBZ. The problem is schematically illustrated for the case of a Dirac-like dispersion cone. In case of single crystal domain (upper panel) a clear band dispersion E(***k***) is detected, reflecting the intersection of the Dirac cone with the measuring plane (E, ***k***) which is defined by the energy axis and momentum direction ***k.*** When energy distribution curves are extracted at K point a sharp peak photoemission feature is found. In case of multiple domain structure with discrete in-plane misalignment (middle panel), additional band dispersions are measured around K point, which results from intersection of the "rotated" Dirac cones with the measuring plane (E, ***k***). Consequently an additional peak appears at the low energy side of the EDC. In case of in plane full rotational disorder (lower panel) a continuous broadening of the band dispersion is introduced in the low kinetic energy side, reflecting the nearly continuous envelope of the band dispersion of the rotated cones on the main measuring plane (E,***k***). Energy broadening is similarly observed in the corresponding EDCs at K point. Importantly, despite the broadening effect, *the measured position of the band edge at K point does not change with respect to the single crystal case.*

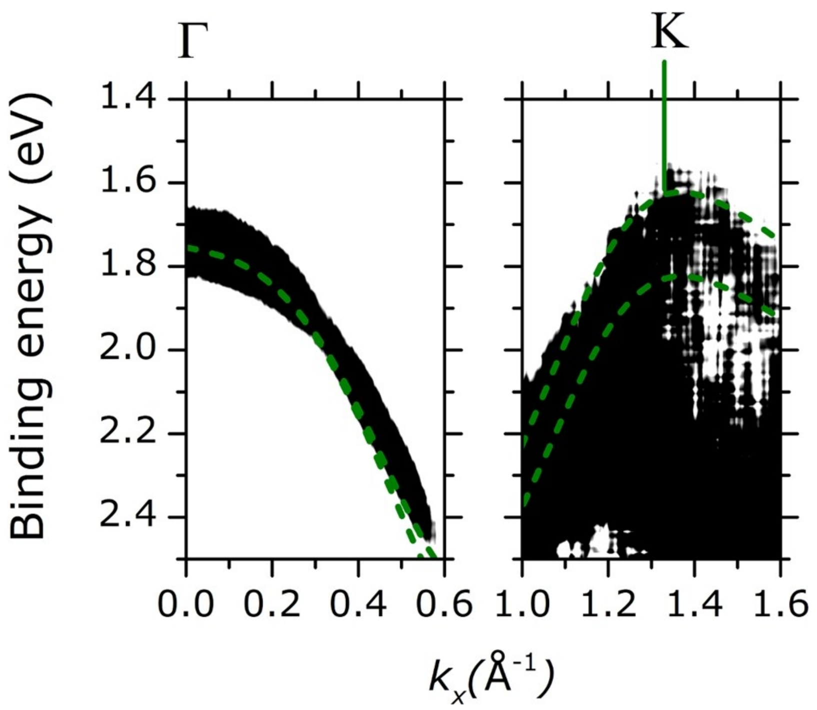

Figure S6. Electronic band dispersion of MoS_2 ML near Γ and K high symmetry point as obtained by second derivative filter of corresponding ARPES data. The colour scale is same as indicated in Figure S4. The contrast is adjusted to maximize the visibility of the experimental band (dark area) near the high symmetry points. Calculated ML band structure along ΓK direction (green dotted line) is also included as for comparison purpose. The experimental and theoretical data are aligned with respect to the binding energy position at Γ point (see main text for related discussion).

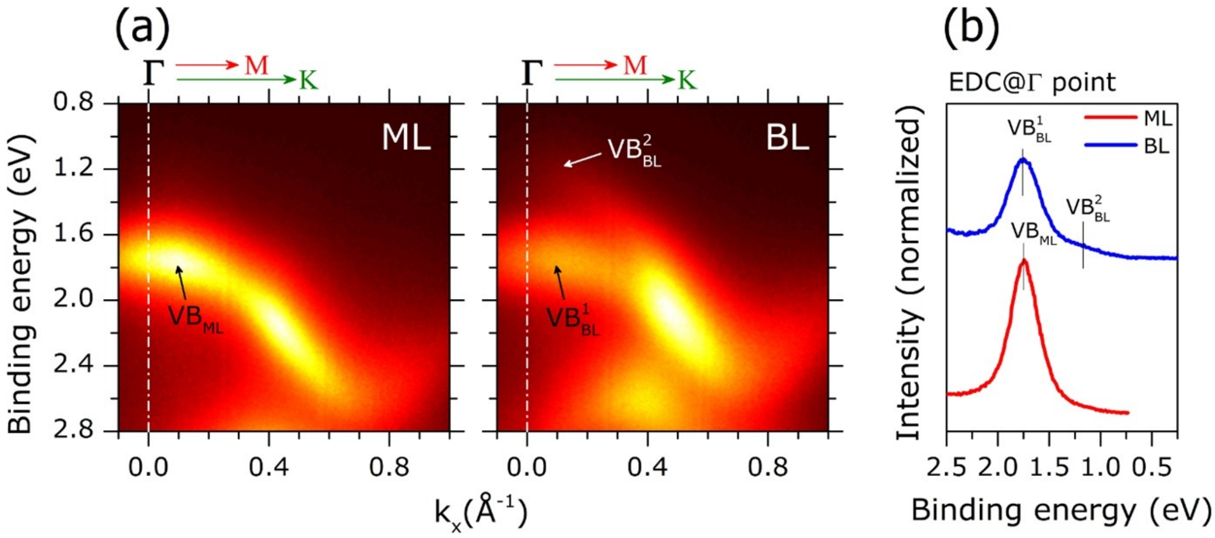

Figure S7. (a) ARPES intensity of MoS_2 ML (left), BL (right) as grown on HOPG substrates and binding energy and momentum component k_x along the (superimposed) ΓM and ΓK high symmetry direction of the SBZ. A clear band splitting at Γ point is observed in passing from the ML (VB_{ML}) to BL thickness (VB^1_{BL}, VB^2_{BL}). (b) Energy distribution curves (EDCs) extracted at Γ point (integration range $\Delta k_x = \pm 0.01$ $Å^{-1}$) as extracted from the ML and BL ARPES data in panel (a). The position of the valence band peaks at Γ point are indicted by vertical bars (see main text for further details).

Supplementary References

[S1] L. Yang, X. Cui, J. Zhang, K. Wang, M. Shen, S. Zeng, S.A. Dayeh, L. Feng and B. Xiang, *Sci. Rep.*, 2014, **4**, 5649

[S2] G. Plechinger, J. Mann, E. Preciado, D. Barroso, A. Nguyen, J. Eroms, C. Schuller, L. Bartels and T. Korn, *Semicond. Sci. Technol.,* 2014, **29**, 064008-16

[S3] S. Mignuzzi *et al., Phys. Rev. B,* 2015*,* **91**, 195411

[S4] H. Li, Q. Zhang, C. C. Ray Yap, B. K.Tay, T.H.T. Edwin,A. Olivier, D. Baillargeat, *Adv. Funct. Mater.* 2012, **22**, 1385–1390
